# Cholera amid COVID-19: Call from three nations; India, Bangladesh, and Nepal

**DOI:** 10.1016/j.amsu.2022.104936

**Published:** 2022-11-14

**Authors:** Abhigan Babu Shrestha, Sumalatha Khatroth, Anannya Malreddy, Farah Abdulkadir Issa, Sajina Shrestha, Shumneva Shrestha

**Affiliations:** a M Abdur Rahim Medical College, Dinajpur, Bangladesh; b Mallareddy Medical College for Women, Suraram, Hyderabad, India; c Cairo University Medical School, Giza, Egypt; d KIST Medical College, Imadol, Patan, Nepal; e Maharajgunj Medical Campus, Institute of Medicine, Tribhuvan University, Kathmandu, Nepal

**Keywords:** Cholera, Nepal, India, Bangladesh, Covid-19

## Abstract

Covid-19 was a major pandemic of the 21st century that flinched away every individual worldwide. The extensive impact of this rapidly spreading deadly virus doomed the health care systems with the unexpected wave wreaked havoc leading to a global health crisis. It has been a high burden on the functioning existing medical system, overloads health professionals, disruption of the medical supply chain. The economy of the nations has been at losses with a significant slowing down in revenue growth over the past 2 years. After taking its toll, drawing away other diseases including cholera. The three developing nations; India, Bangladesh and Nepal, are now at the verge of facing an outbreak of Cholera. It is not surprising to hear cholera in this nation but the fact that its negligence due to Covid-19 pandemic and monkeypox along with a crumbled health system due to the pandemic has made these nations vulnerable for health crisis. Along with this three nations, cholera has made its way to different parts of this globe and it is high time that attention must be drawn towards it as mismanagement could even cause life.

## Highlights

• With ongoing covid-19, cholera has been spotted in many countries including Nepal, India and Bangladesh.
• Possible epidemics could turn the whole country towards health crisis.
• Health care workers, policy makers and public people must stay aware and tackle the potential epidemic.

The major pandemic of the 21st century, named coronavirus disease (COVID-19), was first detected in December 2019 in Wuhan city of China. As of August 2022, this zoonotic coronavirus has been credited with approximately 580,000,000 confirmed cases and 6,400,000 confirmed deaths, making it a global public health crisis [1]. While the unexpected wave of cases wreaked havoc on the well-established health care systems of countries such as the United States, United Kingdom, and Italy, the brunt of this devastation was borne by developing countries such as India, Bangladesh, and Nepal, who were instantaneously overwhelmed. Overpopulation, lack of materials, and broken health care systems resulted in detrimental loss and desolation, having far-reaching effects on the health and wealth of millions of people. With barely sufficient supplies, inadequate sanitation, diminished access to medication, and lack of awareness, they were pushed to their brink by the arrival of this destructive global pandemic. Shortage of medication, life-saving medical equipment, and medical personnel exponentially increased the morbidity and mortality rates, while misinformation and a basic lack of understanding of the situation promoted unnecessary panic among the population. Ensuring the collapse of the already faulty infrastructure and costing the lives and livelihoods of millions of people, leaving them sick, hungry, homeless, or dead, COVID-19 unearthed the deficiencies in our response to a global health crisis. As wave after wave of cases crashed and ravaged these nations over the past two years, what remained of the meager resources were directed towards gaining control of the situation, diverting the much-needed attention away from other diseases that plague these tropical countries, such as cholera.

Cholera is an acute diarrheal disease caused by Vibrio Cholera of the O1 or O139 serogroup, which spreads through contaminated food and water [2]. It first originated in the Ganges delta in 1817 and subsequently spread to seven pandemics killing millions of people across all the continents [2]. V. cholerae is a member of the Vibrionaceae family, a curved, Gram-negative rod that grows best in salt water, and is found in coastal areas and estuaries [3,4]. Based on the O antigen of the liposaccharide, it is classified into more than 200 groups, and out of the O1, and O139 serogroups cause epidemic cholera. O1 serogroup is further classified into subgroups like Inaba and Ogawa based on the O-methylation of the O antigen, and it is classified into Classical and El Tor biotypes based on phenotypic and genetic markers [5].

Cholera affects almost all groups, and it is endemic to many areas in Asia and Africa causing high fatality rates [2]. There are seven recorded pandemics, the first beginning in (1817–1823) in India, and it has spread beyond Asia, with ensuing pandemics in the following years; the second pandemic (1829–1851), the third (1852–1859), the fourth (1863–1879), the fifth (1881–1896), the sixth (1899–1923), and the seventh 1961 to present [6]. There was a unique geographical spread of each pandemic in most of Asia, Africa, Europe, Australia, and the Americas.

India was the first homeland of cholera in 1817 and created an intense epidemic. Despite the vain attempts of governments to contain its fury, cholera spread to several countries. Hence, the first epidemic is called “Asiatic Cholera” [7]. Initially, it spread to Nepal and then headed east, touching southeastern Asia, Indonesia, and Borneo, and finally China and Japan in 1821 and 1822. Currently, cholera is endemic in over 50 countries, but it is under-reported (probably only 5–10% of cases are reported), affecting 3–5 million people each year and killing 100,000 to 120,000 [7]. From 1964 in India, and 1972 in Bangladesh, classical O1 was replaced by El Tor, and the mass migration in Eastern India during the Bangladesh liberation war from 1971 to 1972 is the most important factor. Most people had a sub-clinical infection, and subsequent immunity further protected them from having a clinical disease [6]. The latest seventh pandemic strains of O129 originated from the Bay of Bengal that has spread to distant locations, like India, and Bangladesh. With every pandemic, the frequency, duration, and severity were increasing, and the current pandemic is the longest and gives no evidence of waning. In 2008, Zimbabwe recorded the largest outbreak infecting 100,000 cases and causing death in approximately 3000 people. Subsequently, these numbers were overshadowed by the 2010–2011 cholera outbreak in Haiti infecting approximately 300,000 and causing death in approximately 5000 [5].

With the current conditions of the global economy and Geo-political state, the ongoing COVID-19 pandemic has had a devastating effect, burdening the healthcare systems of developing countries, the limited health resources such as hospital beds, ventilators, oxygen, and vaccine shortages have further pressurized and already struggling health care system. While Southeast Asian countries try to recover from the ongoing effects of the COVID-19 pandemic and deal with the rising monkey pox appearance, a cholera outbreak will further pressurize the already crumbled health care system.

India, Nepal and Bangladesh all with staggering populations have all seen a high number COVID-19 infected people coupled with a substantial number of deaths.

Some of the factors contributing to the high death rate include governmental passivity, lack of preparation, insufficient management, and a pattern of South Asian countries investing heavily in defense expenditures whilst neglecting investments in public health and welfare. Alongside, previously existing impediments such as poverty and the lack of public health infrastructure all add to the underlying cause [8].

In 2021, the World Health Organization (WHO) reported that there will be 1.3 to 4 million cases of cholera. The WHO/UNICEF report that basic infrastructure facilities are lacking for approximately 700 million people and that cholera and enteric diseases are endemic and spreading [3]. UNICEF reports that the sanitation goals in the Millennium Development Program have not been met. Millions of people lack access to clean drinking water, basic sanitation facilities, and sewage treatment systems. Untreated human excreta are a main cause of cholera. According to World Health Organization (WHO) and United Nations Children's Fund (UNICEF) Joint monitoring program (JMP) 2021 reports, 494 million people practice open defecation [9].

“In 2014, the government of India started the Swachh Bharat Mission to provide rural and urban poor communities with subsidies for the construction of toilets [10]. However, not all communities have benefited from scheme. A report of the Joint Monitoring Program for Water Supply, Sanitation and Hygiene by the World Health Organization and UNICEF released in July said around 15% of the country's total population defecates in the open [11].

According to the European Centre for Disease Prevention and Control, currently, most cases of cholera have been reported from Nigeria, India, and Bangladesh [12]. The cases have been escalating in different states of India. Recently, a diarrhea outbreak occurred in Gajhrapurwa locality of Vikasnagar sector 8, where almost 200 people fell sick due to cholera [13]. Similarly, 2 new cases adding in Ahmedabad making a total of 10 cases and 100 suspected cases from Gujarat [12,13]. Even more deleterious consequences due to watery diarrhea is hypovolemic shock leading to death. Death cases were observed in Kashipur block of Rayagada district, where three persons died in less than 24 h whereas an outbreak in Amravati confirmed 5 dead cases due to cholera and affected 181 people [13]. These were mainly associated due to heavy rain falls and flooding in that area associated with unhygienic practicing and consuming contaminated water.

A more grievous situation is seen in Bangladesh, with a reported 495,433 suspected cholera cases and 29 deaths. Most cases were observed from Rohingya Refugee Camp in Cox's Bazar with a total of 33,832 cases [14]. As of 22 weeks 2022, 74 confirmed cholera cases have been noted [15]. Of note, the International Centre for Diarrhoeal Disease Research, Bangladesh (icddr,b) reports daily hospital admission of patients with acute diarrhea exceeding over a thousand in Dhaka [16]. This has made Dhaka a prone area for a potential center for cholera outbreaks. Nevertheless, other major cities like Khulna, Barisal, Chittagong, Mymensingh, Sylhet, Rangpur, and Rajshahi are also on the verge of it [14]. To combat this, the government of Bangladesh has planned to take measures along with the support of WHO, to administer oral cholera vaccines to 2.3 million non-pregnant individuals (older than 1 year) [17].

As of August 7, 2022, a total of 42 cases of Cholera have been reported in Nepal from Kathmandu, Lalitpur, Bhaktapur and Nuwakot districts [18]. Previously, cases were mostly reported from the terai regions of Nepal. However, due to environmental and seasonal change superadded with garbage mismanagement, this year, Kathmandu along with other districts have spotted cases of cholera. Overall, globalization and travel may also have played a role in spreading as occurred previously when the Haiti outbreak occurred in the Caribbean Island with cases migrating from Nepal [19,20]. A single source created a deadly sprout and extensive dissemination along with the clonal expansion of cholera.

As neighboring nations, predictably, if one country experiences an outbreak of a contagious disease, it would not take long for others to follow, as demonstrated in the past. Keeping that in mind, mandating certain practices can drastically reduce the spread of the disease. Recently, precautionary measures have been lax, given the dip in cases a few months back. In light of the current upwards trend of cases, firmly securing borders between countries by strictly monitoring and only allowing people who tested negative for COVID-19 to travel across nations, encouraging vaccination, enforcing mask mandate while in public, and ensuring routine sanitation can lower the contamination rate. Mutual understanding between the officials and the general population is essential to succeed in containing such a disease. According to the recent consensus, India administered more than 2 billion vaccinations [21], while Bangladesh administered upwards of 250 million doses [22], and approximately 69% of Nepal's population are fully vaccinated [23]. All three nations have booster doses available for their population, aiding in dampening the effects of the virus. In addition to that, educating individuals on the Centers for Disease Control's guidelines about the importance of vaccination, the significance of sanitation, maintaining social distancing, and practicing self-isolation and quarantine is paramount to the success of gaining control over this situation [24]. Reinforcing the damaged infrastructure and medical system is crucial to make sure that we are prepared to face any future pandemics, and it is also imperative to ensure that amid such overwhelming demands of the health care system, we are still able to provide basic care for people who are suffering from non-pandemic related medical conditions. Tropical countries, such as the aforementioned three, suffer from endemic outbreaks of cholera regularly, and conditions such as a global pandemic were perfect to instigate such occurrence, amplifying its damaging impact [25]. Therefore, training medical personnel on the latest recommendations and protocols for dealing with such emergencies has long-lasting positive effects and is essential in tackling such a colossal task. Since prevention is always better than cure, proactive measures must be taken to detect outbreaks as soon as they crop up, preventing them from reaching pandemic levels, and minimizing the burden on the health care system. By investigating and correcting any lapses in our response, such devastation can be nipped in the bud. Improving global access to water, sanitation, and hygiene (WASH) is crucial in the efforts of reducing cholera in the Southeast Asia region. The establishment of an antimicrobial sentinel surveillance system across south Asian countries, which includes the identification of cholera, along with plans for use of the WHO stockpile cholera vaccine, steps in the right direction. There is a need for cooperation between policymakers, public health professionals, and workers to create an integrated cholera outbreak response. This is possible with country-wide public health education, cholera vaccine administration, providing water resources and water sanitation education and global assistance [26].

To conclude, the ongoing pandemic COVID-19 and the novel threat of monkeypox has masked the potential outbreak of cholera not only in these three developing nations but around the corners of the globe [27]. This ancient disease has cost lives of millions of people. Nevertheless, these three countries must take into account different factors for preparedness to combat an anticipated cholera outbreak through national and international levels, and other countries parallel to their cases should take action. With a dreadful previous history, this time cholera shouldn't be neglected.

## Ethical approval

Doesn't require.

## Sources of funding

Not sponsored.

## Author contribution

All authors contributed equally.

## Registration of research studies

Name of the registry:
Unique Identifying number or registration ID:
Hyperlink to your specific registration (must be publicly accessible and will be checked):

## Guarantor

Abhigan babu shrestha.

## Consent

N/A.

## Data availability

N/A.

## Provenance and peer review

Not commissioned, externally peer-reviewed.

## Declaration of competing interest

The authors disclose no conflict of interest.
